# Preparation and Enhanced Catalytic Hydrogenation Activity of Sb/Palygorskite (PAL) Nanoparticles

**DOI:** 10.1186/s11671-017-2220-8

**Published:** 2017-07-18

**Authors:** Lin Tan, Muen He, Aidong Tang, Jing Chen

**Affiliations:** 1 School of Chemistry and Chemical Engineering, Central South University, Changsha, 410083 China; 2 Key Laboratory of Palygorskite Science and Applied Technology of Jiangsu Province, Huaiyin Institute of Technology, Huaian, 223003 People’s Republic of China

**Keywords:** Antimony, Palygorskite, Solvothermal synthesis, Catalytic performances

## Abstract

A Sb/palygorskite (PAL) composite was synthesized by a facile solvothermal process and applied in catalytic hydrogenation of *p*-nitrophenol for the first time. It was found that the Sb nanoparticles with the sizes of 2–5 nm were well dispersed on the fiber of PAL, while partial aggregated Sb nanoparticles with sizes smaller than 200 nm were also loaded on the PAL. The Sb/PAL composite with 9.7% Sb mass amounts showed outstanding catalytic performance by raising the *p*-nitrophenol conversion rate to 88.3% within 5 min, which was attributed to the synergistical effect of Sb and PAL nanoparticles facilitating the adsorption and catalytic hydrogenation of *p*-nitrophenol.

**Electronic supplementary material:**

The online version of this article (doi:10.1186/s11671-017-2220-8) contains supplementary material, which is available to authorized users.

## Background

Antimony as a functional material has attracted considerable attention [1, 2]. More recently, reports show that antimony film electrodes offer an unusual characteristic, namely the favorably negative over-voltage of hydrogen evolution [3]. Besides, a new magnetic nanoparticle-supported antimony catalyst is prepared by taking advantage of the interaction between the alkylamines and the Sb nanoparticles, and such technique is applied in wastewater treatment fields [4]. However, the Sb particles were always aggregated together due to their high surface energy, which would hinder their practical application severely. Therefore, the inhibition of particle aggregation remains a thorny problem that waiting to be solved in the next exploration.

Generally, the nanocomposites formed from nanoparticles and various supports demonstrate excellent properties of nanoparticles without the disadvantage of losing any of intrinsic properties of the supports [5–9]. One of the most widely used as supporting materials for the surface modification is clay mineral. The composites by introducing of clay mineral [10–12], such as kaolinite [13, 14], halloysite [15, 16], montmorillonite [17], and sepiolite [18], do not only enhance the dispersion of nanoparticles but also improve the gather of reactants which would produce synergetic effect in the catalytic process and further intensify its catalytic performance [19]. Moreover, the cost of clay mineral is lower than the metal catalyst, which would further reduce the costs of catalysts and facilitate its practical application. Palygorskite (PAL), a species of natural clay mineral with theory formula (Mg,Al,Fe)_5_Si_8_O_20_(OH)_2_(OH_2_)_4_·4H_2_O has been widely applied due to its particular fiber-liked morphology [20–22] which endowed unique properties, such as larger surface area [23], nontoxicity [24], and excellent adsorption capacity [25]. Owing to such particular properties, PAL is used as adsorbents [26, 27], catalysts, and catalyst supports [19]. For example, the modified PAL shows superior adsorption capacities than raw PAL [28, 29]. Moreover, Y_2_O_3_ functionalized PAL was used as an adsorbent and exhibited potential applications in wastewater treatment [25]. In conclusion, the nanocomposites formed from the combination of PAL and nanoparticles show extraordinary catalytic properties of nanoparticles, and its great surface area allows an increase in the catalyst sensitivity. In our previous study, rich antimony hollow Sb_2_Se_3_ sphere particles demonstrate an excellent catalytic property for the hydrogenation of *p*-nitrophenol [30, 31]. However, the effect of antimony on the process of *p*-nitrophenol hydrogenation remains unclear. Therefore, a series of Sb/PAL hybrid composites with different Sb contents are prepared, and their catalytic performance of *p*-nitrophenol hydrogenation also undergoes investigation. The synthesized strategy is dispersing Sb particles on the PAL fiber surface via a facile solvothermal process and creating more reaction active sites to enhance its catalytic property.

## Methods

The PAL was purchased from Xuyi, China. In a typical synthesis process of Sb/PAL composites with the antimony mass content of 9.7% (marked as 9.7% Sb/PAL), antimony potassium tartrate (0.124 g), and PAL (0.456 g) were mixed in 55 ml of ethanol/water solution with the volume ratio of 40:15, then under continuously stirring for 30 min. Subsequently, NaBH_4_ (0.030 g) was dissolved in 15 ml of deionized water. Afterwards, the solution was dropwise added into above mixture within 10 min. Later, it was transferred into an 80 ml of Teflon-lined autoclave. From that moment, it stayed sealed and maintained at 180 °C for 5 h. Later, the as-synthesized products were washed with ethanol and deionized water for three times, then collected and held at 80 °C in an oven for 6 h. Finally, the products were grinded for further characterization and test. Also, Sb/PAL composites with different loaded amounts of Sb were fabricated in the above similar method through controlling the amounts of antimony potassium tartrate and sodium borohydride while keeping PAL mass constant.

The X-ray diffraction analysis (XRD), scanning electron microscopy (SEM), energy dispersive spectrometer (EDS), transmission electron microscopy (TEM), and high-resolution transmission electron microscopy (HRTEM) were tested as previous literature [30]. The UV–vis spectrum was detected on SHIMADZU UV-2450 spectrophotometer, and the spectrum range was 205–500 nm. The Fourier transform infrared analysis (FTIR) was carried out on a Bruker VERTEX-70 spectrometer with KBr pellets between 4000 and 400 cm^−1^. The inductively coupled plasma emission spectrometry (ICP) was tested on Perkin Elmer Optima 5300.

The catalytic activity of as-fabricated products was tested for the *p*-nitrophenol catalytic hydrogenation to *p*-aminophenol in the presence of NaBH_4_. In a typically catalytic procedure, *p*-nitrophenol aqueous solution (100 μL 0.025 mol/L) was mixed with 20 ml of deionized water, and the following procedures remain the same as our previous works [30].

## Results and Discussion

**Fig. 1:**
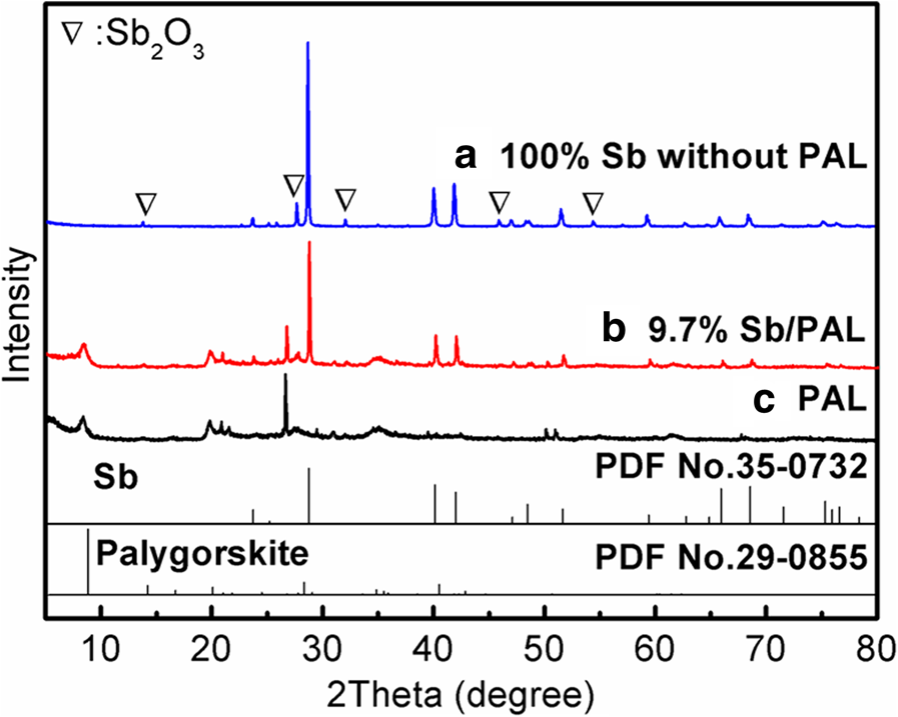
XRD patterns of (**a**) 100% Sb without PAL, (**b**) 9.7% Sb/PAL, and (**c**) PAL sample

The XRD patterns of the as-prepared products were displayed in Fig. 1. The main diffraction peaks of the sample of 100% Sb with no PAL addition (Fig. 1 (a)) could be indexed to antimony (PDF No.35-0732). Meanwhile, tiny amounts of Sb_2_O_3_ (PDF No.05-0534) could also be found in the figure, which may be produced through the redox reaction on the antimony surface. Besides, the diffraction peaks of the raw palygorskite (Fig. 1 (c)) were in accordance with the palygorskite (PDF No. 29–0855). Meanwhile, the diffraction peak at 2*θ* = 26.6° was attributed to the quartz [19]. After Sb particle combined with PAL fiber (Fig. 1 (b)), the corresponding diffraction peaks were referred to palygorskite (PDF No.29-0855) and antimony (PDF No.35-0732). These results implied that the Sb particles have loaded on the palygorskite and formed the Sb/PAL hybrid composite.

**Fig. 2:**
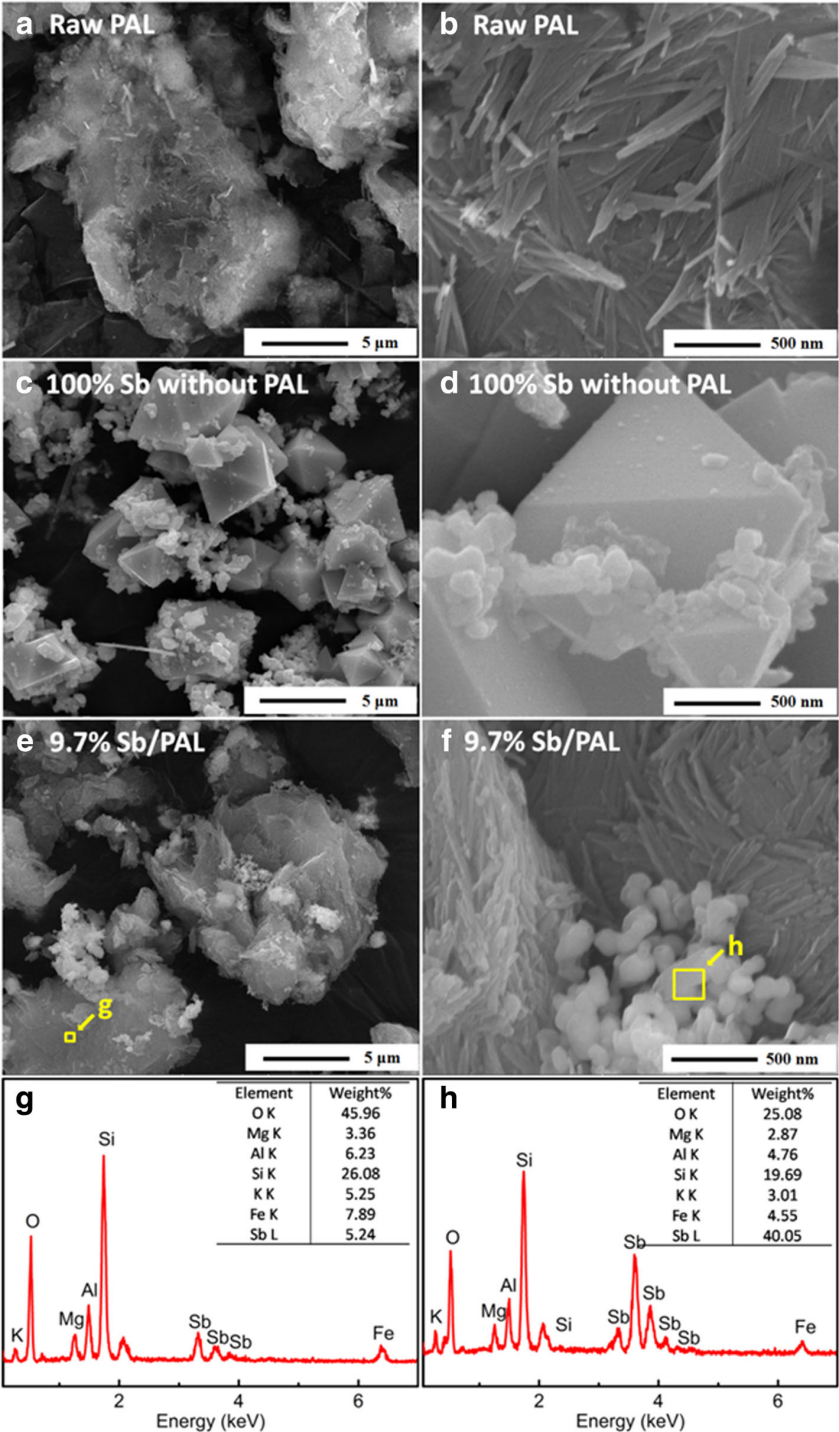
SEM images of **a**, **b** PAL, **c**, **d** 100% Sb without PAL, and **e**, **f** 9.7% Sb/PAL sample and **g**, **h** EDS patterns of 9.7% Sb/PAL

The SEM images of palygorskite in Fig. 2a, b showed that numerous fibers aggregated into bulk crystal bundles with flat or sheet structures due to the strong interaction among the fibers of palygorskite [32]. It was found that the PAL fiber was about 40 nm in diameter and several hundred nanometers in length. For the 100% Sb without PAL sample as Fig. 2c displayed, several octahedral-shaped particles were surrounded by numerous irregular particles. The size of the octahedral edge was about 1 μm while the size of irregular particle was larger than 100 nm (Fig. 2d). Besides, the irregular particles were aggregated together severely. For 9.7% Sb/PAL displayed in Fig. 2e, f, after the Sb particles anchored on the PAL fiber, some particles with a diameter under 200 nm was aggregated together on the fibers surface while no large-sized Sb particles similar to the octahedral shape displayed in Fig. 2c was found. This phenomenon indicated that the PAL played a key role in limiting the growth of Sb nanoparticles, despite it was still partly aggregated together.

The EDS analysis of different regions in 9.7% Sb/PAL composites was carried out to investigate the Sb nanoparticle distribution, and the results were shown in Fig. 2g, h. For the flat region signed in Fig. 2e, the mass contents of Sb were only 5.24% which was lower than the theoretical amounts of 9.7%. But for the aggregated region marked in Fig. 2f, the mass amounts of Sb was increased from the theoretical values of 9.7% to the realistic values of 40.05%. The above results indicated that the obtained part of Sb nanoparticles were not as well ordered; monodispersed Sb particles as one might be expected on the PAL surface, possibly due to the fact that PAL is difficult to be well dispersed.

**Fig. 3:**
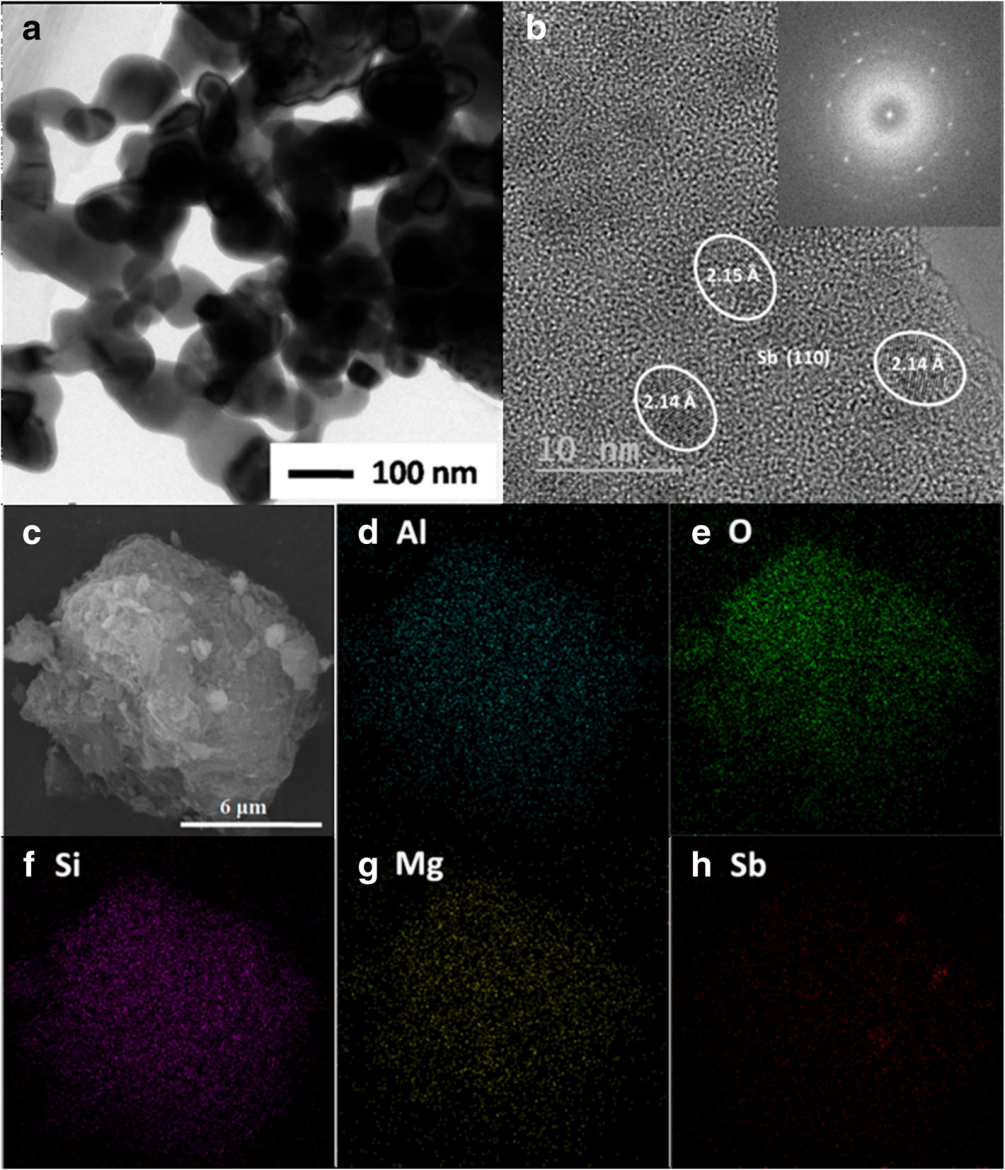
**a** TEM image, **b** HRTEM image, the *inserted image* is the SAED pattern, and **c** *–* **h** the elemental map of 9.7% Sb/PAL

TEM and HRTEM images of the 9.7% Sb/PAL were tested and displayed in Fig. 3a, b respectively. The diameters of the aggregated spherical Sb particles were about 100 nm, which were corresponded to the SEM results. The monodispersed Sb particles of the size 2–5 nm found on Fig. 3b were widely distributed on the PAL surface, and the *d*-spacing of the Sb particle was sized as 0.214 nm, which indexed to the (110) plane of Sb (PDF No.35-0732) as well. The selected area electron diffraction (SAED) pattern of the sample displayed in Fig. 3b using inserted figure showed several diffraction ring patterns and diffraction spots, demonstrating that the Sb/PAL hybrid composites were polycrystalline. The element distribution of 9.7% Sb/PAL composites was shown in Fig. 3c–h. The Al, O, Si, Mg, and Sb elements were distributed homogeneously throughout the composites except for Sb element formed three small regions of uneven distribution. This phenomenon further indicated that the Sb nanoparticles were widely distributed on the PAL surface while exhibiting a partial uneven distribution. However, the result of HRTEM clearly demonstrated that the monodispersed Sb particle with the size of 2–5 nm was widely distributed on the PAL surface.

**Fig. 4:**
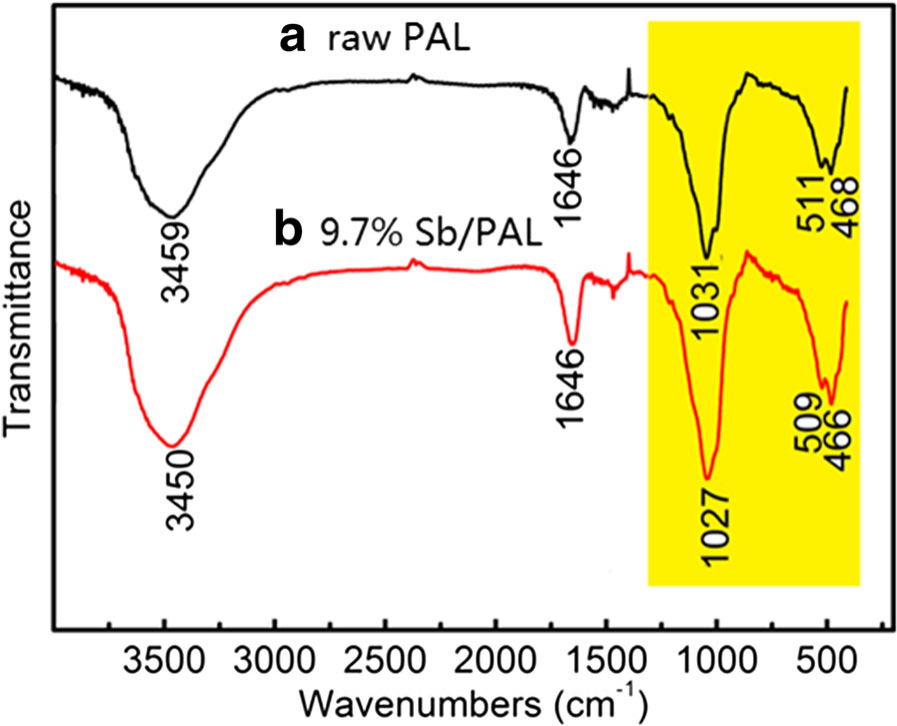
FTIR spectra of (**a**) PAL and (**b**) 9.7% Sb/PAL

In order to investigate the interaction between Sb nanoparticle and palygorskite, the FTIR spectrum of raw palygorskite and 9.7% Sb/PAL composites were displayed in Fig. 4. For the raw PAL (Fig. 4 (a)), the band in 3459 and 1646 cm^−1^ were attributed to the stretching vibrations of hydroxyl group and the bending vibration of adsorbed water respectively [33, 34]. Meanwhile, the wide band around 1031 cm^−1^ was related to the stretch vibrations of silicon–oxygen bond [20]. And the band at 468 and 511 cm^−1^ was attributed to the silicon–oxygen–silicon bending vibration [35]. After the Sb particles anchored on PAL fibers (Fig. 4 (b)), although no new absorbance band appeared, the related absorbance band of PAL was shifted to lower wave numbers as marked in yellow highlight in Fig. 4, such as 1027 cm^−1^ of the stretch vibrations of silicon–oxygen bond and 466 and 509 cm^−1^ relating to the silicon–oxygen–silicon bending vibration. This phenomenon implied the existence of the chemical interaction between Sb and the silicon hydroxyl group on the PAL surface, weakening the bond of silicon–oxygen–silicon. Such similar effects have been reported by Peng et al. [11].

**Fig. 5:**
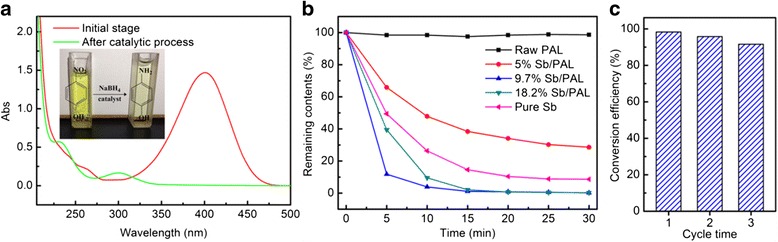
**a** UV–vis absorption spectra of *p*-nitrophenol aqueous in the presence of 9.7% Sb/PAL composites catalyst, **b** catalytic activities of different sample, and **c** the recyclability of the 9.7% Sb/PAL hybrid composites

The catalytic performance of as-prepared sample was tested for the *p*-nitrophenol catalytic reduction to *p*-aminophenol in the presence of NaBH_4_. To identify the catalytic production, the *p*-nitrophenol aqueous solution was tested by UV–vis spectrophotometer in the range of 205 to 500 nm, and the results were showed in Fig. 5a. After the catalytic reaction, the maximum peak at 400 nm was decreased close to zero, while the position at 300 nm have a noticeable increment, indicating that *p*-nitrophenol have converted into *p*-aminophenol [36].

The catalytic performance of several different samples were tested, and the results were displayed in Fig. 5b. The contents of *p*-nitrophenol ions were kept almost constant for the pure PAL, indicating that the pure PAL had no contribution to the catalytic process, therefore, implied that the hydrogenation process would not occur in the absence of the catalyst. Meanwhile, for the pure Sb, the *p*-nitrophenol catalytic efficiency reached to 91.4% within 30 min. Upon adding 5% Sb/PAL composites to this system, the conversion rate of *p*-nitrophenol ions was measured at 71.5% within a time period of 30 min. With the loaded amounts of Sb increased to 9.7 and 18.2%, the conversion rates were significantly raised to 98.2 and 97.3% respectively, which was higher than the 100% Sb with no PAL sample at the rate of 91.4%. More importantly, it was noteworthy that the catalytic efficiency of 9.7% Sb/PAL composites stands at 88.3% within 5 min, which was about 1.7 times higher than the efficiency of 50.6% achieved within 5 min by using only 100% Sb without PAL. The pure Sb (without PAL) demonstrates higher *p*-nitrophenol conversion (91.4%) than in the presence of 5% Sb/PAL (71.5%) that is because the Sb content is very low. As Additional file 1: Figure S1 displayed, the peak intensity of Sb in 5% Sb/PAL composites was comparatively low while that of Sb_2_O_3_ was high. This results indicated that the number of Sb particles is also a main factor for the reduction of *p*-nitrophenol.

**Fig. 6:**
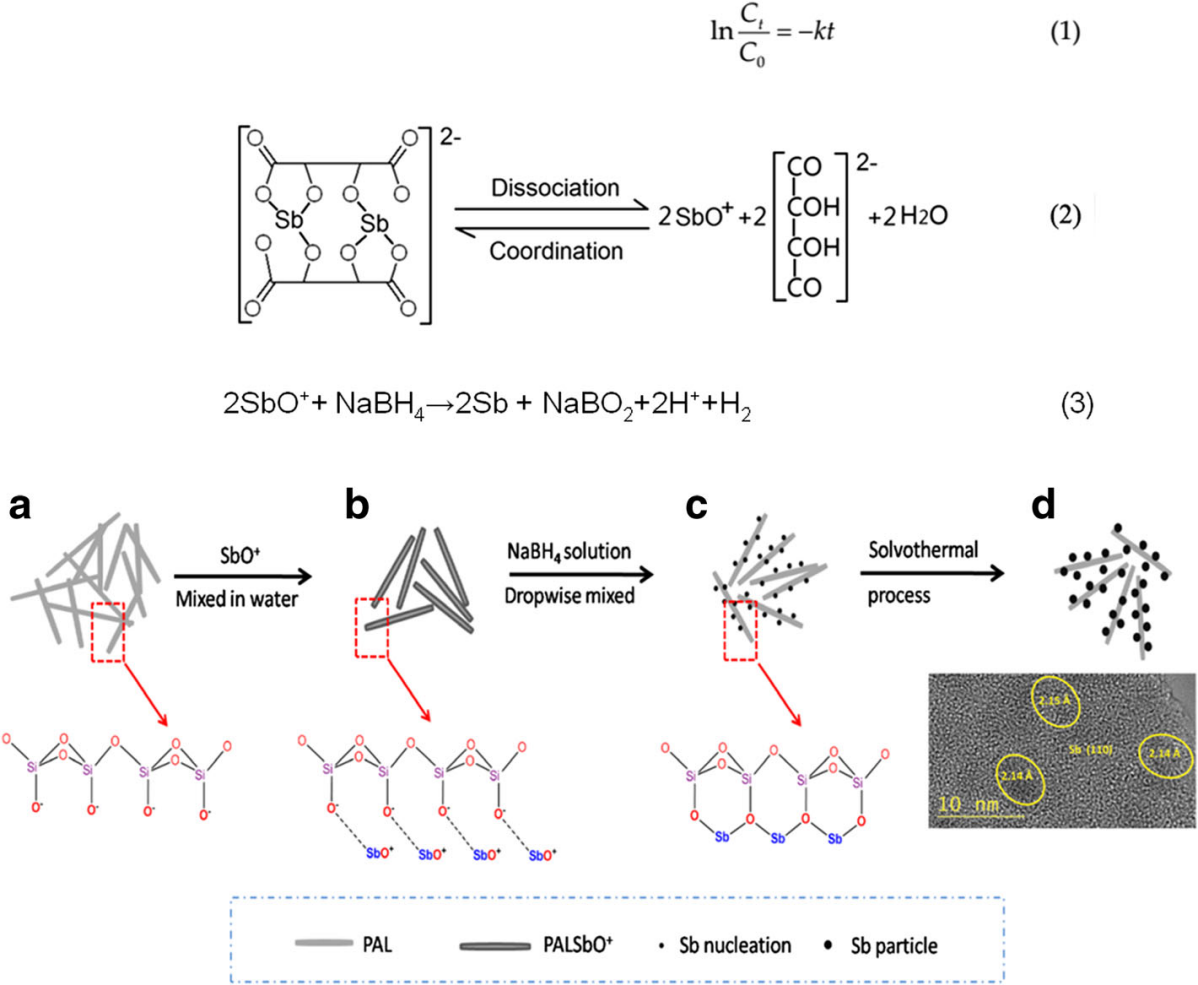
The pseudo first-order kinetics equation, corresponding chemical reaction equtions and Schematic illustration of the Sb/PAL composites fabrication

As we have known, the hydrogenation reaction of *p*-nitrophenol follow a pseudo first-order kinetics equation displayed in Eq. (1) (Fig. 6) when the amount of NaBH_4_ was far higher than the amount of *p*-nitrophenol [37]. Hence, to further reveal the catalytic performance of the sample, we calculated the apparent reaction rate constant of 9.7% Sb/PAL sample and collected some other rate constant recorded by previous literature, and the data was given in Additional file 2: Table S1. The reaction rate constant of 9.7% Sb/PAL sample could reach to 0.420 min^−1^ which showed an excellent catalytic performance.

1 $$ \mathrm{In}\frac{C_t}{C_0}=\hbox{-} k t $$

To investigate the stability of the Sb/PAL composites, the reusability experiment of the 9.7% Sb/PAL was tested and the result was shown in Fig. 5c. It was observed that the conversion efficiency of *p*-nitrophenol within 30 min was 91.6% after three cycles. The catalytic results demonstrated that the Sb/PAL composites perform an excellent catalytic hydrogenation with a good reusability, which was attributed to the high dispersion of Sb nanoparticles on the palygorskite fiber, providing a more active site; the similar effect was also found in TiO_2_/halloysite composites [38].

Based on the above experimental results, a possible fabrication mechanism of the Sb/PAL composites was proposed. Firstly, palygorskite was a fibrous clay mineral with a structure consisting of short and alternating inverted 2:1 sheets or ribbons. These ribbons were of an average width (along the Y direction) of two linked tetrahedral chains. The tetrahedral sheet was continuous across the ribbons but with apices pointing up and down in adjacent ribbon [22]. These silica tetrahedral ribbons had abundant Si–OH groups, which could adsorb and hold the cations such as Fe^3+^, Ni^2+^ ions [19, 39] and SbO^+^ ions as well. Secondly, the complex dissociation equilibrium of antimony tartrate complex ions was shown as Eq. (2). Although the antimony potassium tartrate was a stable coordination compound, it could provide a slow complex dissociation way to form a few of SbO^+^ ions, hence, controlling the rate of the reactive process as well. Thus SbO^+^ ions were gradually adsorbed on the surface of PAL fiber with abundant Si–OH. The similar effect was found in the Pd/kaolinite composites [40].

Thirdly, when the NaBH_4_ aqueous solution was dropwise introduced into the above system, the numbers of SbO^+^ ions would decrease due to the Sb particles forming according to the redox reaction Eq. (3) which would further lead to the dissociation of antimony tartrate complex ions. Besides, the newly formed H^+^ ions originated from Eq. (3) also benefited to the releasing of SbO^+^ ions because of the acidic effect which would further improve the Sb precursor combining with the PAL [41]. Therefore, with the reducing agent NaBH_4_ being introduced into the system, the initial Sb nanoparticle attached on the PAL surface in situ via the Si–OH sited on the silica tetrahedral ribbons.

Finally, Sb/PAL composites with highly dispersed Sb nanoparticles were fabricated via the solvothermal process. Furthermore, those dissociative SbO^+^ ions were reduced, forming some aggregated Sb particles among the layers of PAL. In reverse, if the PAL fiber was absent, the particles would aggregate together and forming large sized Sb particles in a octahedral shape due to their high surface energy. Fig. 6 presents the schematic illustration of the Sb/PAL composites fabrication. The PAL rod served as a template for the growth of Sb nanoparticles and effectively inhibited the aggregation of Sb particles. Although it was found that some Sb nanoparticles were still partly aggregated together since PAL is difficult to be well dispersed, the size of Sb particles clearly declined below 200 nm. In addition, the Sb/PAL hybrid composite showed an excellent catalytic property, ascribed to its abundant interface between the Sb nanoparticles and PAL, which helped significantly in promoting *p*-nitrophenol adsorption and facilitating the catalytic hydrogenation of *p*-nitrophenol.

## Conclusions

The Sb/PAL nanocomposites were synthesized through a facile solvothermal process through using natural palygorskite as a base. According to the characterized results, the PAL fiber could effectively inhibit the Sb nanoparticle aggregation. In addition, the composites were tested for the *p*-nitrophenol catalytic hydrogenation process. The 9.7% Sb/PAL composites showed excellent catalytic performance and its *p*-nitrophenol conversion efficiency reaching 88.3% within 5 min, which was about 1.7 times more efficient than using only 100% Sb without PAL adding. Therefore, the tested composites prove outstanding properties and offer excellent potential in future practical catalytic applications.

## Additional files

Additional file 1: Figure S1. XRD pattern of 5% Sb/PAL (PNG 72 kb)

Additional file 2: Table S1. Comparison of the catalytic performances of Sb/PAL and other available catalysts obtained from previous literature. (DOCX 181 kb)

## Abbreviations

(Mg,Al,Fe)_5_Si_8_O_20_(OH)_2_(OH_2_)_4_·4H_2_O: Palygorskite
EDS: Energy dispersive spectrometer
FTIR: Fourier transform infrared spectroscopy
HRTEM: High-resolution transmission electron microscopy
ICP: Inductively coupled plasma emission spectrometry
KBr: Potassium bromide
NaBH_4_: Sodium borohydride
PAL: Palygorskite
Pd: Palladium
PDF: Powder diffraction files
SAED: Selected area electron diffraction
Sb: Antimony
Sb_2_O_3_: Antimonous oxide
SEM: Scanning electron microscope
TEM: Transmission electron microscopy
XRD: X-ray diffraction
Y_2_O_3_: Yttrium oxide
