# Supplementary figures and images for: Preparation and Enhanced Catalytic Hydrogenation Activity of Sb/Palygorskite (PAL) Nanoparticles
Source: Nanoscale Res Lett. 2017 Jul 18;12:460. doi: 10.1186/s11671-017-2220-8 (PMC5515725; doi:10.1186/s11671-017-2220-8)

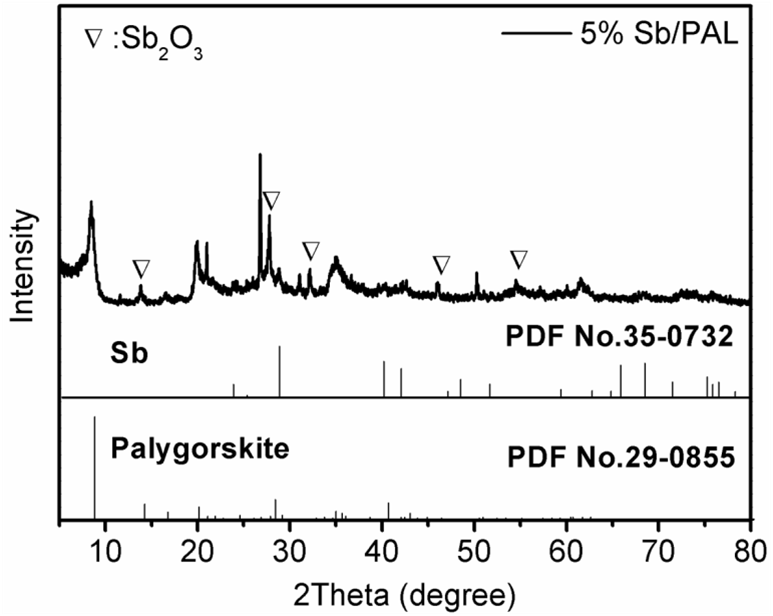

Supplement: Supplementary file 1 — XRD pattern of 5% Sb/PAL (PNG 72 kb) [file 11671_2017_2220_MOESM1_ESM.png]
