# Supplementary material for: Preparation and Enhanced Catalytic Hydrogenation Activity of Sb/Palygorskite (PAL) Nanoparticles
Source: Nanoscale Res Lett. 2017 Jul 18;12:460. doi: 10.1186/s11671-017-2220-8 (PMC5515725; doi:10.1186/s11671-017-2220-8)
Supplement: Supplementary file 2 — Comparison of the catalytic performances of Sb/PAL and other available catalysts obtained from previous literature. (DOCX 181 kb) [file 11671_2017_2220_MOESM2_ESM.docx]

Supplementary Information

Preparation and enhanced catalytic hydrogenation activity of Sb/palygorskite(PAL) nanoparticles

Lin Tan^a^, Muen He^a^, Aidong Tang ^* a^, Jing Chen ^* b^

^a^ *School of Chemistry and Chemical* Engineering, Central *South University, Changsha 410083, China*

^b^ *Key Laboratory of Palygorskite Science and Applied Technolog yof Jiangsu Province, Huaiyin Institute of Technology, Huaian 223003, PR China*

*Author, email:*

Lin Tan (lin_tan199106@163.com), Muen He (459062711@qq.com), Aidong Tang (adtang@csu.edu.cn), Jing Chen (chenjing6910@163.com)

*^*^ Corresponding author, Email: adtang@csu.edu.cn, chenjing6910@163.com*

*Tel.: +86/731/88879616, Fax: +86/731/88879616.*

**Table S1** Comparison of the catalytic performances of Sb/PAL and other available catalysts obtained from previous literature.

| Catalyst | k(min^-1^) | Catalyst loading | Initial p-NP | NaBH_4_ | Reference |
| --- | --- | --- | --- | --- | --- |
| Pt-Au/RGO | 0.575 | 30 μl, 2 g/L | 2.7 mL, 0.1 mM | 0.3 mL, 0.1M | [[1](#_ENREF_1)] |
| Gold nanorods | 1.02 | 10 μl, 3 nM | 60 μl, 5 mM | 3 mL, 0.05M | [[2](#_ENREF_2)] |
| Ni_0.52_Sb_0.48_Sb/SBA-15 | 0.227 | 1 ml, 50 g/L | 1 ml, 0.1 mM | 1 ml, 0.06 M | [[3](#_ENREF_3)] |
| NiSb | 0.216 | 1 ml, 0.1 g/L | 1 ml, 0.1 mM | 1 mL, 0.06 M | [[4](#_ENREF_4)] |
| Sb_2_Se_3_/TiO_2_ | 0.035 | electrode | 2 ml, 0.1 mM | 1 mL, 0.1 M | [[5](#_ENREF_5)] |
| Sb/Sb_2_Se_3_ | 0.325 | 20 mg | 20 ml, 0.15 mM | 10 ml, 0.1 M | [[6](#_ENREF_6)] |
| MoS_2_/PMMT | 0.723 | 1 ml, 1 g/L | 50ml, 0.12mM | 0.136 g | [[7](#_ENREF_7)] |
| Sb/PAL | 0.420 | 20 mg | 20 ml, 0.125 mM | 10 ml, 0.1 M | This work |


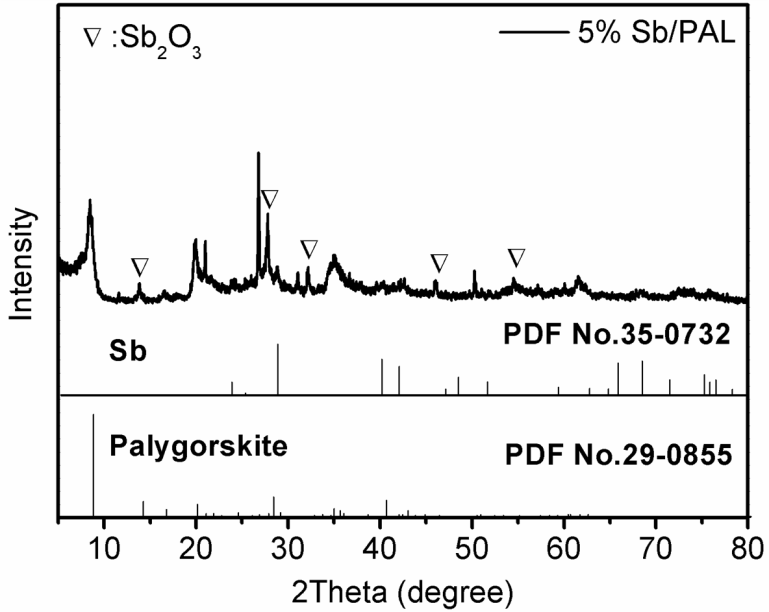


**Fig. S1** XRD pattern of 5% Sb/PAL

**References**

1. Ye W, Yu J, Zhou Y, Gao D, Wang D, Wang C, Xue D (2016) Green synthesis of Pt-Au dendrimer-like nanoparticles supported on polydopamine-functionalized graphene and their high performance toward 4- nitrophenol reduction. APPLIED CATALYSIS B-ENVIRONMENTAL 181

2. Xiong W, Sikdar D, Yap LW, Guo P, Premaratne M, Li X, Cheng W (2016) Matryoshka-caged gold nanorods: Synthesis, plasmonic properties, and catalytic activity. Nano Research 9:415-423

3. Marakatti VS, Peter SC (2016) Nickel–antimony nanoparticles confined in SBA-15 as highly efficient catalysts for the hydrogenation of nitroarenes. New Journal of Chemistry 40:5448-5457

4. Shanbogh PP, Peter SC (2013) Low cost nano materials crystallize in the NiAs structure type as an alternative to the noble metals in the hydrogenation process. RSC Advances 3:22887-22890

5. Tang A, Long M, He Z (2014) Electrodeposition of Sb_2_Se_3_ on TiO_2_ nanotube arrays for catalytic reduction of p-nitrophenol. Electrochimica Acta 146:346-352

6. Tang AD, Long M, Liu P, Tan L, He Z (2014) Morphologic control of Sb-rich Sb_2_Se_3_ to adjust its catalytic hydrogenation properties for p-nitrophenol. RSC Advances 4:57322-57328

7. Peng K, Fu L, Yang H, Ouyang J, Tang A (2016) Hierarchical MoS_2_ intercalated clay hybrid nanosheets with enhanced catalytic activity. Nano Research 10:570-583
